# Instance-based concept learning from multiclass DNA microarray data

**DOI:** 10.1186/1471-2105-7-73

**Published:** 2006-02-16

**Authors:** Daniel Berrar, Ian Bradbury, Werner Dubitzky

**Affiliations:** 1 School of Biomedical Sciences, University of Ulster at Coleraine, Cromore Road, Northern Ireland, UK

## Abstract

**Background:**

Various statistical and machine learning methods have been successfully applied to the classification of DNA microarray data. Simple instance-based classifiers such as nearest neighbor (NN) approaches perform remarkably well in comparison to more complex models, and are currently experiencing a renaissance in the analysis of data sets from biology and biotechnology. While binary classification of microarray data has been extensively investigated, studies involving multiclass data are rare. The question remains open whether there exists a significant difference in performance between NN approaches and more complex multiclass methods. Comparative studies in this field commonly assess different models based on their classification accuracy only; however, this approach lacks the rigor needed to draw reliable conclusions and is inadequate for testing the null hypothesis of equal performance. Comparing novel classification models to existing approaches requires focusing on the significance of differences in performance.

**Results:**

We investigated the performance of instance-based classifiers, including a NN classifier able to assign a degree of class membership to each sample. This model alleviates a major problem of conventional instance-based learners, namely the lack of confidence values for predictions. The model translates the distances to the nearest neighbors into 'confidence scores'; the higher the confidence score, the closer is the considered instance to a pre-defined class. We applied the models to three real gene expression data sets and compared them with state-of-the-art methods for classifying microarray data of multiple classes, assessing performance using a statistical significance test that took into account the data resampling strategy. Simple NN classifiers performed as well as, or significantly better than, their more intricate competitors.

**Conclusion:**

Given its highly intuitive underlying principles – simplicity, ease-of-use, and robustness – the *k*-NN classifier complemented by a suitable distance-weighting regime constitutes an excellent alternative to more complex models for multiclass microarray data sets. Instance-based classifiers using weighted distances are not limited to microarray data sets, but are likely to perform competitively in classifications of high-dimensional biological data sets such as those generated by high-throughput mass spectrometry.

## Background

### Motivation

Being crucial to diagnostic and prognostic applications, a plethora of methods have been brought to bear on microarray data classification in the field of cancer research [1-3]. Microarray data analysis is beset by the 'curse of dimensionality' (a.k.a. small-*n*-large-*p *problem) [4]. This problem relates to the high dimensionality, *p*, i.e., the number of gene expression values measured for a single sample, and the relatively small number of biological samples, *n*.

There is a growing number of publications on comparative studies trying to elucidate the performance of various classifiers for microarray data sets. However, the conclusions that can be drawn from these studies are often limited because of one or more of the following reasons.

(1) The study involves only binary classification tasks [5].

(2) The study does not involve a complete re-calibration of all model parameters in each learning phase [6].

(3) The study does not incorporate an external cross-validation to avoid gene selection bias [7].

(4) The study makes inappropriate use of clustering techniques for classification tasks [8].

(5) The study assesses the differences in performance based on 'orphaned' accuracy measures (e.g., observed cross-validation error rates).

Many comparative studies include data sets involving binary problems only. One of the first studies in this field compared a nearest neighbor model, support vector machines, and boosted decision stumps on three binary microarray data sets related to cancer [9]. The recent study by Krishnapuram *et al*. benchmarked their model against a variety of statistical and machine learning methods using two cancer microarray data sets involving a binary classification task [10]. Tasks involving multiple classes, however, are considered substantially more challenging. Li *et al*. [11] and Yeang *et al*. [12] highlighted the importance of multiclass methodologies in this context.

It is common practice to assess microarray classifiers using data resampling strategies such as bootstrapping and cross-validation strategies. Dudoit *et al*. have highlighted the importance of model re-calibration in each cross-validation fold [6]; however, comparative studies do not always include a complete parameter recalibration [8].

It is crucial that feature selection or weighting is performed only on the learning set and not on the test set. Otherwise, the estimation of the model's generalization ability will be overly optimistic [7]. Whereas this caveat may not have received due attention in early microarray studies, most recent comparative studies include an external cross-validation phase intended to avoid the selection bias.

One of the most common pitfalls in the analysis of microarray data analysis is the use of clustering methods for classification tasks [8]. Clustering methods are unsupervised methods that do not take into account the class labels. The number of class-discriminating genes is usually small compared with the number of non-discriminating genes. The pair-wise distances that clustering methods compute do not necessarily reflect the influence of the discriminating genes. Hence, the resulting clusters may not be related to the phenotypes at hand. Different clustering methods can reveal different insights in the data by providing different clusters, all of which may be of interest – there is generally no 'right' or 'wrong' clustering result.

Finally, a critical problem in the aforementioned comparative studies is that these models are commonly assessed based on monolithic accuracy measures, frequently devoid of suitable confidence intervals for the true error rates (or alternatively, the true prediction accuracy). Comparing classification error rates or confidence intervals is limited in terms of the conclusions that can be drawn when comparing differences in performance. It is crucial that a comparative study assesses these differences based on suitable significance tests that also take into account the adopted resampling strategy. In an ideal world with unlimited training and test data, the comparison of classifiers would be straightforward. However, in practical settings, the number of available cases is limited, and particularly small in the context of microarray data. Therefore, the classifiers are usually compared based on their performance on resampled training and test sets. The sampling procedure introduces a random variation in the sampled data sets, which must be controlled by the statistical test [13]. For example, the classification performance of the same method can be different, depending on whether leave-one-out cross-validation, ten-fold cross-validation, or bootstrapping is adopted for data set sampling. The statistical test should conclude that two models perform significantly differently if and only if their error rate would be different, on average, when trained on a training set of a given fixed size and tested on all cases of the population of interest [13]. This is essentially the aim of comparative studies: Do the observed differences in performance provide sufficient evidence to conclude that the models perform significantly differently, or can we not exclude the possibility (with reasonably confidence) that this difference may be due to chance alone or to the random variation introduced by the sampling strategy? This question should guide the formulation of the null hypothesis. In general, this implies that for a randomly drawn learning set of fixed size and according to a fixed probability distribution, two models will have the same error rate on a test set that is also randomly drawn from the population under investigation, and all random draws are made according to the same probability distribution [13]. Note, that a 95%-*confidence interval for an estimate *(e.g., the true prediction accuracy) is completely different from a 95%-*confidence level for the difference *of two estimates (e.g., the difference between the prediction accuracy of model *A *and *B*). Therefore, it should be noted explicitly that it is logically inadequate to use the derived confidence intervals for assessing whether there is a significant *difference *in performance of the classifiers. This fact is well-established in the statistical literature, but may not have received sufficient attention in many comparative studies.

Somorjai *et al*. [4] identified the following key features of classifiers for microarray data: *Robustness *(i.e., high generalization ability and insensitivity with respect to outliers) and the *simplicity *of a model. A model (*i*) should be easy to implement and use, and (*ii*) its outputs should be easy to interpret. In particular in biomedical applications, we claim that such classifiers should also be able to provide a suitable measure of confidence for the predictions they make. One way of representing such a confidence measure could be a degree of class membership with respect to the predicted class. In such a framework, a sample may belong to any class with a certain degree. This is often represented by the unit interval: A value of 0 indicating complete non-membership and a value of 1 indicating complete compliance with the predefined class in questions. Any value within the interval indicates a partial class membership. Providing such a value of 'confidence' for classifications can serve two purposes, (*i*) optimizing the model's calibration in the learning phase, and (*ii*) the rejection of low-confidence classifications in the test phase.

### Overview of nearest neighbor classifiers

Comparative studies involving various classifiers and microarray data sets have revealed that instance-based learning (a basic form of memory-based or case-based reasoning) approaches such as nearest neighbor methods perform remarkably well compared with more intricate models [14,15]. A *k*-nearest neighbor (*k*-NN) classifier is based on an instance-based learning concept, which is also referred to as lazy learning. In contrast to eager methods, which apply rule-like abstractions obtained from the learning instances, lazy methods access learning instances at application time, i.e., the time when a new case is to be classified. A nearest neighbor classifier determines the classification of a new sample on the basis of a set of *k *similar samples found in a database containing samples with known classification. Challenges of the *k*-NN approach include (a) the relative weighting of features, (b) the choice of a suitable similarity method, (c) the estimation of the optimal number of nearest neighbors, and (d) a scheme for combining the information represented by the *k *nearest neighbors.

In its simplest implementation, *k*-NN computes a measure of similarity between the test case and all pre-classified learning cases. The test case is then classified as a member of the same class as the most similar case [11]. In this simple scenario only one, the most similar case, is finally selected for calling the class, the parameter *k *is set to 1. A more elaborate variant of *k*-NN involves cross-validation procedures that determine an optimal number, *k*_*opt*_, of nearest neighbors; usually, *k*_*opt *_> 1. The test case is classified based on a majority vote among the *k*_*opt *_nearest neighbors [16]. For example, in leave-one-out cross-validation, each hold-out case is classified based on *k *∈ {1, 2, ..., *k*_*max*_} neighbors. That integer *k *that minimizes the cumulative error is *k*_*opt*_. For more details and extensions to the *k*-NN classifier, see for instance [5,16-18], and references therein.

### Paper outline

Motivated by the recent success stories of nearest neighbor methods [14,15,19,20], we investigated a model of a *k*-nearest neighbor classifier based on a weighted-voting of normed distances [5,16]. This classifier outputs a degree of class membership for each case **x**, 0 ≤ p^
 MathType@MTEF@5@5@+=feaafiart1ev1aaatCvAUfKttLearuWrP9MDH5MBPbIqV92AaeXatLxBI9gBaebbnrfifHhDYfgasaacH8akY=wiFfYdH8Gipec8Eeeu0xXdbba9frFj0=OqFfea0dXdd9vqai=hGuQ8kuc9pgc9s8qqaq=dirpe0xb9q8qiLsFr0=vr0=vr0dc8meaabaqaciaacaGaaeqabaqabeGadaaakeaacuWGWbaCgaqcaaaa@2E25@ (*C *| **x**) ≤ 1. Wang *et al*. used fuzzy *c*-means clustering for deriving fuzzy membership values, which they used as a confidence measure for microarray data classification [21]. Recently, Asyali and Alci applied fuzzy *c*-means clustering for classifying microarray data of two classes [22]. In contrast to the models of Wang *et al*. [21] and Asyali and Alci [22], the *k*-NN model in the present study does not rely on unsupervised clustering approaches for deriving fuzzy class membership values.

This paper focuses on a simple and intuitive model, the *k*-nearest neighbor based on distance weighting, for the classification of multiclass microarray data and aims at addressing the aforementioned key limitations of previous comparative studies in this field. We apply the distance-weighted *k*-NN to three well-studied, publicly available microarray data sets, one based on cDNA chips and two on Affymetrix oligonucleotide arrays, and compare the classification performance with support vector machines (SVMs), decision tree C5.0 (DT), artificial neural networks (multiplayer perceptrons, MLPs), and 'classic' nearest neighbor classifiers (1-NN, 3-NN, and 5-NN) that are based on majority voting. The 5-NN is not applied to the NCI60 data set because of the small number of cases per class. Using a ten-fold repeated random subsampling strategy, we assess the models' classification performance based on a 0–1 loss function, i.e., a loss of 0 for each correct classification and a loss of 1 for each misclassification. To allow for a 'crisp' classification using *k*-NN, a case **x **is classified as member of class *C *for which p^
 MathType@MTEF@5@5@+=feaafiart1ev1aaatCvAUfKttLearuWrP9MDH5MBPbIqV92AaeXatLxBI9gBaebbnrfifHhDYfgasaacH8akY=wiFfYdH8Gipec8Eeeu0xXdbba9frFj0=OqFfea0dXdd9vqai=hGuQ8kuc9pgc9s8qqaq=dirpe0xb9q8qiLsFr0=vr0=vr0dc8meaabaqaciaacaGaaeqabaqabeGadaaakeaacuWGWbaCgaqcaaaa@2E25@ (*C *| **x**) is maximal. We do not consider the rejection of low-confidence classifications. The statistical significance of the differences in performance is assessed using a parametric test, the variance-corrected resampled paired *t*-test [23].

## Results

### Classification results

Let *f *denote the observed fraction of correctly classified test cases and let *p *denote the true prediction accuracy of the model. Let the total number of test cases be *M*. For deriving a (1 - *α*)100%-confidence interval for the true prediction accuracy *p*, we obtain Equation (1) by the de Moivre-Laplace limit theorem (assuming that the binomial distribution of the correctly classified cases can be approximated by the standard normal):

|f−p|f(1−f)/M<Φ−1(1−12α)     (1)
 MathType@MTEF@5@5@+=feaafiart1ev1aaatCvAUfKttLearuWrP9MDH5MBPbIqV92AaeXatLxBI9gBaebbnrfifHhDYfgasaacH8akY=wiFfYdH8Gipec8Eeeu0xXdbba9frFj0=OqFfea0dXdd9vqai=hGuQ8kuc9pgc9s8qqaq=dirpe0xb9q8qiLsFr0=vr0=vr0dc8meaabaqaciaacaGaaeqabaqabeGadaaakeaadaWcaaqaaiabcYha8jabdAgaMjabgkHiTiabdchaWjabcYha8bqaamaakaaabaGaemOzayMaeiikaGIaeGymaeJaeyOeI0IaemOzayMaeiykaKIaei4la8Iaemyta0ealeqaaaaakiabgYda8iabfA6agnaaCaaaleqabaGaeyOeI0IaeGymaedaaOGaeiikaGIaeGymaeJaeyOeI0YaaSWaaSqaaiabigdaXaqaaiabikdaYaaaiiGakiab=f7aHjabcMcaPiaaxMaacaWLjaWaaeWaaeaacqaIXaqmaiaawIcacaGLPaaaaaa@4B5C@

with Φ(•) being the standard normal cumulative distribution function and *z *= Φ^-1^(1 - 1/2*α*), e.g., *z *= 1.96 for 95% confidence. Solving Equation (2) for *p *gives Equation (2):

p=(f+z22M±zfM−f2M+z24M2)/(1+z2M)     (2)
 MathType@MTEF@5@5@+=feaafiart1ev1aaatCvAUfKttLearuWrP9MDH5MBPbIqV92AaeXatLxBI9gBaebbnrfifHhDYfgasaacH8akY=wiFfYdH8Gipec8Eeeu0xXdbba9frFj0=OqFfea0dXdd9vqai=hGuQ8kuc9pgc9s8qqaq=dirpe0xb9q8qiLsFr0=vr0=vr0dc8meaabaqaciaacaGaaeqabaqabeGadaaakeaacqWGWbaCcqGH9aqpdaqadaqaaiabdAgaMjabgUcaRmaalaaabaGaemOEaO3aaWbaaSqabeaacqaIYaGmaaaakeaacqaIYaGmcqWGnbqtaaGaeyySaeRaemOEaO3aaOaaaeaadaWcaaqaaiabdAgaMbqaaiabd2eanbaacqGHsisldaWcaaqaaiabdAgaMnaaCaaaleqabaGaeGOmaidaaaGcbaGaemyta0eaaiabgUcaRmaalaaabaGaemOEaO3aaWbaaSqabeaacqaIYaGmaaaakeaacqaI0aancqWGnbqtdaahaaWcbeqaaiabikdaYaaaaaaabeaaaOGaayjkaiaawMcaaiabc+caVmaabmaabaGaeGymaeJaey4kaSYaaSaaaeaacqWG6bGEdaahaaWcbeqaaiabikdaYaaaaOqaaiabd2eanbaaaiaawIcacaGLPaaacaWLjaGaaCzcamaabmaabaGaeGOmaidacaGLOaGaayzkaaaaaa@54FA@

Table 1 shows the 95%-confidence intervals for the true prediction accuracy of the models, averaged over the ten test sets.

**Table 1:** 95%-confidence intervals for the true prediction accuracy (in %).

	NCI60	ALL	GCM
*k*-NN	72.10 ± 7.07	**77.85 ± 2.43**	74.39 ± 3.88
1-NN	72.10 ± 7.07	76.96 ± 2.46	74.80 ± 3.86
3-NN	63.65 ± 7.59	77.76 ± 2.43	71.49 ± 4.02
5-NN	-	77.76 ± 2.43	71.49 ± 4.02
SVM	**78.60 ± 6.44**	77.58 ± 2.44	**75.83 ± 3.81**
DT	63.00 ± 7.62	68.86 ± 2.71	64.88 ± 4.25
MLP	61.70 ± 7.68	70.20 ± 2.67	55.17 ± 4.43

**Figure 1:**
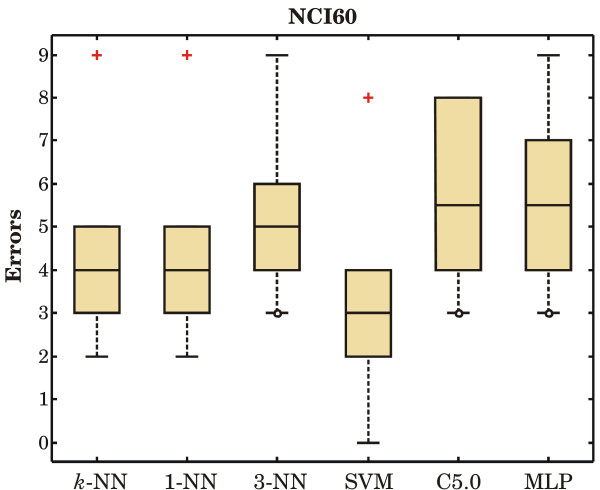
**Prediction errors on the NCI60 data set**. The total number of misclassified cases in all ten folds are: 41 by distance-weighted *k*-NN, 41 by 1-NN, 54 by 3-NN, 31 by SVM, 55 by DT, and 57 by MLP.

**Figure 2:**
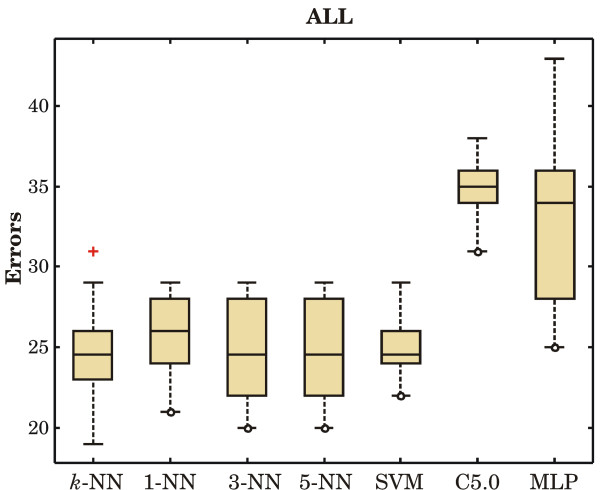
**Prediction errors on the ALL data set**. The total number of misclassified cases in all ten folds are: 247 by distance-weighted *k*-NN, 257 by 1-NN, 248 by 3-NN, 248 by 5-NN, 250 by SVM, 348 by DT, and 333 by MLP.

**Figure 3:**
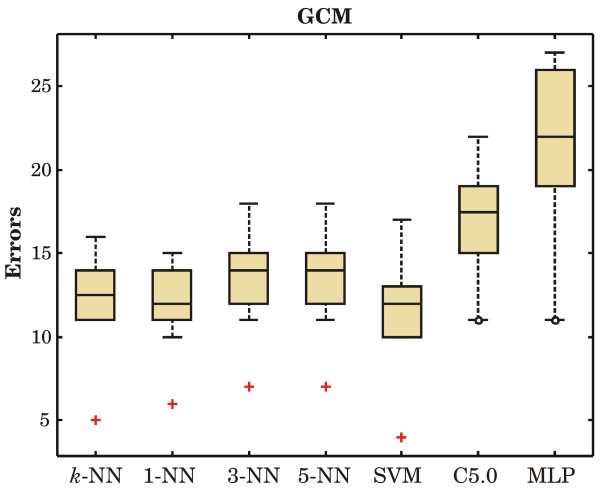
**Prediction errors on the GCM data set**. The total number of misclassified cases in all ten folds are: 122 by distance-weighted *k*-NN, 120 by 1-NN, 136 by 3-NN, 136 by 5-NN, 115 by SVM, 168 by DT, and 215 by MLP.

Figures 1 to 3 show the boxplots of prediction errors.

Equation (3) provides a (1 - *α*)100%-confidence interval for the differences in prediction errors.

1k∑i=1k|εAi−εBi|±tk−1,12α×SE     (3)
 MathType@MTEF@5@5@+=feaafiart1ev1aaatCvAUfKttLearuWrP9MDH5MBPbIqV92AaeXatLxBI9gBaebbnrfifHhDYfgasaacH8akY=wiFfYdH8Gipec8Eeeu0xXdbba9frFj0=OqFfea0dXdd9vqai=hGuQ8kuc9pgc9s8qqaq=dirpe0xb9q8qiLsFr0=vr0=vr0dc8meaabaqaciaacaGaaeqabaqabeGadaaakeaadaWcaaqaaiabigdaXaqaaiabdUgaRbaadaaeWbqaamaaemaabaacciGae8xTdu2aaSbaaSqaaiabdgeabjabdMgaPbqabaGccqGHsislcqWF1oqzdaWgaaWcbaGaemOqaiKaemyAaKgabeaaaOGaay5bSlaawIa7aaWcbaGaemyAaKMaeyypa0JaeGymaedabaGaem4AaSganiabggHiLdGccqGHXcqScqWG0baDdaWgaaWcbaGaem4AaSMaeyOeI0IaeGymaeJaeiilaWYaaSWaaWqaaiabigdaXaqaaiabikdaYaaaliab=f7aHbqabaGccqGHxdaTcqqGtbWucqqGfbqrcaWLjaGaaCzcamaabmaabaGaeG4mamdacaGLOaGaayzkaaaaaa@560D@

where *k *= 10 is the number of folds, *ε*_*Ai *_is the observed error of model *A *in the *i*^th ^fold, *t*_9, 0.025 _= 2.26 for 95% confidence, and SE is the standard error as shown in the denominator in Equation 4. Table 2 shows the 95%-CI for the differences in prediction errors.

**Table 2:** 95%-confidence intervals for the differences in prediction errors.

		1-NN	3-NN	5-NN	SVM	DT	MLP
NCI60	*k*-NN	0	1.30 ± 0.09	-	1.40 ± 0.16	2.0 ± 0.21	1.60 ± 0.16
	1-NN	-	1.30 ± 0.09	-	1.40 ± 0.16	2.0 ± 0.21	1.60 ± 0.16
	3-NN	-	-	-	2.30 ± 0.16	1.30 ± 0.22	1.10 ± 0.15
	SVM	-	-	-	-	2.60 ± 0.23	2.60 ± 0.21
	DT	-	-	-	-	-	1.80 ± 0.21
ALL	*k*-NN	2.60 ± 0.05	0.90 ± 0.02	0.90 ± 0.02	2.90 ± 0.06	10.10 ± 0.06	8.80 ± 0.08
	1-NN	-	2.90 ± 0.05	2.90 ± 0.05	1.50 ± 0.03	9.10 ± 0.03	7.80 ± 0.10
	3-NN	-	-	0	3.20 ± 0.06	10.0 ± 0.06	8.70 ± 0.08
	5-NN	-	-	-	3.20 ± 0.06	10.0 ± 0.06	8.70 ± 0.08
	SVM	-	-	-	-	9.80 ± 0.02	8.30 ± 0.09
	DT	-	-	-	-	-	5.30 ± 0.10
GCM	*k*-NN	0.40 ± 0.02	2.20 ± 0.07	2.20 ± 0.07	2.10 ± 0.08	4.60 ± 0.05	9.30 ± 0.09
	1-NN	-	2.20 ± 0.06	2.20 ± 0.06	2.10 ± 0.08	4.80 ± 0.06	9.50 ± 0.09
	3-NN	-	-	0.00	2.90 ± 0.09	3.80 ± 0.09	7.90 ± 0.09
	5-NN	-	-	-	2.90 ± 0.09	3.80 ± 0.09	7.90 ± 0.09
	SVM	-	-	-	-	5.70 ± 0.12	10.00 ± 0.13
	DT	-	-	-	-	-	4.70 ± 0.11

The apparent 'best' performers in the present study are the support vector machines with a classification accuracy of 78.60 ± 6.44% on the NCI60 data set and an accuracy of 75.83 ± 3.81% on the GCM data set. However, as we will show later, this result does not necessarily imply that the differences in performance between nearest neighbor models and the support vector machines are statistically significant.

On the ALL data set, the *k*-NN achieved the highest classification accuracy of 77.85 ± 2.43%. The results of the present study do not match up with the results that Yeoh *et al*. reported [3], i.e., a best average test set accuracy of 98.67%. How can this discrepancy be explained? First, the present study assessed the models' performance in a 10-fold random subsampling procedure that entailed ten splits of learning and test sets. The study of Yeoh *et al*., on the other hand, comprised one split only (i.e., single hold-out approach) [3], so that the achieved classification accuracies may not reflect the true performance of their models. Second, the classification task in the present study includes all ten classes, whereas Yeoh *et al*. focused on the classification results for the six molecularly distinct classes [3].

### Analysis of differences in performance

Assume that in each fold, *N *cases are used for learning and *M *cases are used for testing. Let the number of folds be *k*. Let the difference of proportion of misclassified cases be *p*_*i *_= *p*_*Ai *_- *p*_*Bi*_, with *i *= 1..*k *and *p*_*Ai *_= *m*_*Ai*_/*M*, with *m*_*Ai *_the number of errors on the *i*^th ^test set comprising *M *cases (*p*_*Bi *_and *m*_*Bi *_analogous). Let the average of *p*_*i *_over the *k *folds be p¯=k−1∑i=1kpi
 MathType@MTEF@5@5@+=feaafiart1ev1aaatCvAUfKttLearuWrP9MDH5MBPbIqV92AaeXatLxBI9gBaebbnrfifHhDYfgasaacH8akY=wiFfYdH8Gipec8Eeeu0xXdbba9frFj0=OqFfea0dXdd9vqai=hGuQ8kuc9pgc9s8qqaq=dirpe0xb9q8qiLsFr0=vr0=vr0dc8meaabaqaciaacaGaaeqabaqabeGadaaakeaacuWGWbaCgaqeaiabg2da9iabdUgaRnaaCaaaleqabaGaeyOeI0IaeGymaedaaOWaaabmaeaacqWGWbaCdaWgaaWcbaGaemyAaKgabeaaaeaacqWGPbqAcqGH9aqpcqaIXaqmaeaacqWGRbWAa0GaeyyeIuoaaaa@3C3D@. The estimated variance of the *k *differences is s2=(k−1)−1∑i=1k(pi−p¯)2
 MathType@MTEF@5@5@+=feaafiart1ev1aaatCvAUfKttLearuWrP9MDH5MBPbIqV92AaeXatLxBI9gBaebbnrfifHhDYfgasaacH8akY=wiFfYdH8Gipec8Eeeu0xXdbba9frFj0=OqFfea0dXdd9vqai=hGuQ8kuc9pgc9s8qqaq=dirpe0xb9q8qiLsFr0=vr0=vr0dc8meaabaqaciaacaGaaeqabaqabeGadaaakeaacqWGZbWCdaahaaWcbeqaaiabikdaYaaakiabg2da9iabcIcaOiabdUgaRjabgkHiTiabigdaXiabcMcaPmaaCaaaleqabaGaeyOeI0IaeGymaedaaOWaaabmaeaacqGGOaakcqWGWbaCdaWgaaWcbaGaemyAaKgabeaakiabgkHiTiqbdchaWzaaraGaeiykaKYaaWbaaSqabeaacqaIYaGmaaaabaGaemyAaKMaeyypa0JaeGymaedabaGaem4AaSganiabggHiLdaaaa@462C@. The statistic for the variance-corrected resampled paired *t*-test is then given as shown in Equation (4).

Tc=p¯(k−1+M/N)s2∼tk−1     (4)
 MathType@MTEF@5@5@+=feaafiart1ev1aaatCvAUfKttLearuWrP9MDH5MBPbIqV92AaeXatLxBI9gBaebbnrfifHhDYfgasaacH8akY=wiFfYdH8Gipec8Eeeu0xXdbba9frFj0=OqFfea0dXdd9vqai=hGuQ8kuc9pgc9s8qqaq=dirpe0xb9q8qiLsFr0=vr0=vr0dc8meaabaqaciaacaGaaeqabaqabeGadaaakeaacqWGubavdaWgaaWcbaGaem4yamgabeaakiabg2da9maalaaabaGafmiCaaNbaebaaeaadaGcaaqaaiabcIcaOiabdUgaRnaaCaaaleqabaGaeyOeI0IaeGymaedaaOGaey4kaSIaemyta0Kaei4la8IaemOta4KaeiykaKIaem4Cam3aaWbaaSqabeaacqaIYaGmaaaabeaaaaGccqWI8iIocqWG0baDdaWgaaWcbaGaem4AaSMaeyOeI0IaeGymaedabeaakiaaxMaacaWLjaWaaeWaaeaacqaI0aanaiaawIcacaGLPaaaaaa@47A5@

This statistic obeys approximately Student's *t *distribution with *k *- 1 degrees of freedom. The only difference to the standard *t *statistic is that the factor 1/*k *in the denominator has been replaced by 1/*k *+ *M*/*N*. In cross-validation and repeated random subsampling, the learning sets *L*_*i *_necessarily overlap; in repeated random subsampling, the test sets may overlap as well. Hence, the individual differences *p*_*i *_are not independent from each other. Due to these violations of the basic independence assumptions, the standard paired *t*-test cannot be applied here. Empirical results show that the corrected statistic improves on the standard resampled *t*-test; the Type I error is drastically reduced [23,24]. For *k *= 10 folds, the null hypothesis of equal performance between two classifiers can be rejected at *α *= 0.05 if |*T*_*c*_| > *t*_9, 0.025 _= 2.26.

We applied the following six classifiers to the NCI60 data set: *k*-NN, 1-NN, 3-NN, SVMs, DT, and MLP. The 5-NN is applied to the ALL and GCM data set but not to the NCI60 data set because of the small number of cases per class.

Based on the variance-corrected resampled paired *t*-test, we cannot reject the null hypothesis of equal performance between *k*-NN and the SVMs on the NCI60 data set (*P *= 0.38). Hence, the support vector machines did not perform significantly better than *k*-NN on this data set. The smallest *p*-value is *P *= 0.06 for the comparison between SVMs and 3-NN, which does not allow for the rejection of the null hypothesis of equal performance.

On the ALL data set, we observe no statistically significant difference in performance between *k*-NN and the support vector machines (*P *= 0.92), but between *k*-NN and the decision tree (*P *= 0.007). The support vector machines performed significantly better than the decision tree (*P *= 1.67 × 10^-6^), but not significantly better than the multilayer perceptron (*P *= 0.11). The support vector machines did not perform significantly better than 1-NN (*P *= 0.63), 3-NN (*P *= 0.95), or 5-NN (*P *= 0.95). It might seem surprising that the *p*-value is smaller for the comparison support vector machines vs. decision tree (*P *= 1.67 × 10^-6^) than *k*-NN vs. decision tree (*P *= 0.007) despite the fact that the confidence intervals for the true prediction accuracy of the support vector machines and decision tree are 'closer to each other'. However, we note that a 95%-confidence interval for an estimate (here, the true prediction accuracy of a model) is completely different from a 95%-confidence level for the *difference *of two estimates (here, the difference between the accuracies of two models).

On the GCM data set, the difference in performance between *k*-NN and the decision tree is significant (*P *= 0.003) as well as between *k*-NN and the multilayer perceptron (*P *= 0.001). There is no significant difference between *k*-NN and the support vector machines (*P *= 0.70).

In summary, on all three data sets, there was no statistically significant difference in performance between the decision tree and the multilayer perceptron. On all data sets, there was no statistically significant difference between *k*-NN and the support vector machines. The *k*-NN outperformed the decision tree on both the ALL and the GCM data set, and the *k*-NN outperformed the MLP on the GCM data set.

When a comparative study comprises *n *classifiers, a total of κ = 1\2 *n*(*n *- 1) pairwise comparisons are possible. The *α *of each individual test is the comparison-wise error rate, while the family-wise error rate (a.k.a. overall Type I error rate), *α*_κ_, is made up of the κ individual comparisons. To control the family-wise error rate, different approaches are possible, for example Bonferroni's correction for multiple testing, which sets *α*/κ as comparison-wise error rate. The corrected comparison-wise error rates are then *α *= 0.05/15 = 0.0033 for the NCI60 data set and *α *= 0.05/21 = 0.0024 for the ALL and GCM data set. Taking this correction into account, the *p*-value for the difference in performance between *k*-NN and DT on the ALL data set, *P *= 0.007, is to be compared with *α *= 0.0024, and hence the null hypothesis of equal performance cannot be rejected anymore. However, Bonferroni's method is known to be conservative. We are currently investigating various approaches for addressing this problem in the context of multiclass microarray data.

## Discussion

The design of this investigation takes into account the caveats of comparative studies by including a complete model re-calibration in each learning phase, an external cross-validation strategy, and by assessing the models' performance based on significance tests rather than relying on accuracy measures. The presented *k*-NN classifier alleviates a major problem of the 'classic' nearest neighbor models, i.e., the lack of confidence values for the predictions. We derived a degree of class membership without the need for clustering methods. The model is simple, intuitive, and both its implementation and application are straightforward. Despite its simple underlying principles, *k*-NN performed as well as or even better than established more intricate machine learning methods.

In the present study, the classification results with confidence values had to be converted into crisp classifications based on the maximal p^
 MathType@MTEF@5@5@+=feaafiart1ev1aaatCvAUfKttLearuWrP9MDH5MBPbIqV92AaeXatLxBI9gBaebbnrfifHhDYfgasaacH8akY=wiFfYdH8Gipec8Eeeu0xXdbba9frFj0=OqFfea0dXdd9vqai=hGuQ8kuc9pgc9s8qqaq=dirpe0xb9q8qiLsFr0=vr0=vr0dc8meaabaqaciaacaGaaeqabaqabeGadaaakeaacuWGWbaCgaqcaaaa@2E25@, because we assessed and compared the models using a 0–1 loss function. The degrees of class memberships have been used as guidance for model calibration in the learning phase, but these degrees could also be used for the rejection of low-confidence classifications in the test phase. This potential of the *k*-NN has not been exploited in the present study. Different quantitative criteria are possible for comparing classifiers, for example, the quadratic loss function or the informational loss function that both take into account the classifiers' confidence in the predictions, or the costs that are involved for false positive and false negative predictions. This is of particular interest for applications in the biomedical context. In an ongoing study, we compare and assess various models that are able to generate confidence values for the classification. Here, we are interested in the critical assessment of classifiers that take into account the confidences, which can also entail the rejection of classification decisions. Also, the problem of adjusting the error rate for multiple testing needs further work.

## Conclusion

Instance-based learning approaches are currently experiencing a renaissance for classification tasks involving high-dimensional data sets from biology and biotechnology. The *k*-NN performed remarkably well compared to its more intricate competitors. A significant difference in performance between *k*-NN and support vector machines could not be observed. Viewed from an Occam's razor perspective, we doubt that more intricate classifiers should necessarily be preferred over simple nearest neighbor approaches. This is particularly relevant in practical biomedical scenarios where life scientists have a need to understand the concepts of the methods used in order to fully accept them.

## Methods

### Data

The NCI60 data set comprises gene expression profiles of 60 human cancer cell lines of various origins (both derived from solid and non-solid tumors) [1]. Scherf *et al*. [29] used Incyte cDNA microarrays that included 3,700 named genes, 1,900 human genes homologous to those of other organisms, and 4,104 ESTs of unknown function but defined chromosome map location. The data set includes nine different cancer classes: Central nervous system (6 cases), breast (8 cases), renal (8 cases), non-small cell lung cancer (9 cases), melanoma (*8 cases*), prostate (2 cases), ovarian (6 cases), colorectal (7 cases), and leukemia (6 cases). The background-corrected intensity values of the remaining genes are log_2_-transformed prior to analysis.

The ALL data set comprises the expression profiles of 327 pediatric acute lymphoblastic leukemia samples [3]. The diagnosis of ALL was based on the morphological evaluation of bone marrow and on an antibody test. Based on immunophenotyping and cytogenetic approaches, six genetically distinct leukemia subtypes have been identified: B lineage leukemias *BCR-ABL *(15 cases), *E2A-PBX *(27 cases), *TEL-AML *(79 cases), rearrangements in the *MLL *gene on chromosome 11q23 (20 cases); *hyperdiploid karyotype *(> 50 chromosomes, 64 cases); and T lineage leukemias (43 cases). In total, 79 cases could not be assigned to any of the aforementioned groups; these samples were assigned to the group *Others*. This group comprises four subgroups: *Hyperdiploid 47–50 *(23 cases), *Hypodiploid *(9 cases), *Pseudodiploid *(29 cases), and *Normaldiploid *(18 cases). The present study follows the data pre-processing as described in [3], supplementary online material.

Ramaswamy *et al*. investigated the expression profiles in 198 specimens (190 primary tumors and eight metastatic samples) of predominantly solid tumors using Hu6800 and Hu35KsubA Affymetrix chips containing 16,063 oligonucleotide probe sets [2]. The GCM data set comprises 14 cancer classes in total: Breast adenocarcinomas (12 cases), prostate adenocarcinomas (14 cases), lung adenocarcinomas (12 cases), colorectal adenocarcinomas (12 cases), lymphoma (22 cases), bladder transitional cell carcinomas (11 cases), melanomas (10 cases), uterine adenocarcinomas (10 cases), leukemia (30 cases), renal cell carcinomas (11 cases), pancreatic adenocarcinomas (11 cases), ovarian adenocarcinomas (12 cases), pleural mesotheliomas (11 cases), and carcinomas of the central nervous system (20 cases).

### Study design

#### Dimension reduction and feature selection

We decided to focus on two widely used methods to address the high-dimensionality problem: Principal component analysis (PCA) based on singular value decomposition [26] and the signal-to-noise (S2N) metric [27]. PCA reduces dimensionality and redundancy by mapping the existing genes onto a smaller set of 'combined' genes or 'eigengenes' [28]. The S2N metric (a.k.a. Slonim's *P*-metric) is a simple, yet powerful approach for assigning weights to genes, thus permitting analysis to focus on a subset of important genes [2,15,27]. For the *i*^th ^gene and the *j*^th ^class, the signal-to-noise weight *w*_*ij *_is determined as shown in Equation (5):

wij=mij−m′ijsij+s′ij     (5)
 MathType@MTEF@5@5@+=feaafiart1ev1aaatCvAUfKttLearuWrP9MDH5MBPbIqV92AaeXatLxBI9gBaebbnrfifHhDYfgasaacH8akY=wiFfYdH8Gipec8Eeeu0xXdbba9frFj0=OqFfea0dXdd9vqai=hGuQ8kuc9pgc9s8qqaq=dirpe0xb9q8qiLsFr0=vr0=vr0dc8meaabaqaciaacaGaaeqabaqabeGadaaakeaacqWG3bWDdaWgaaWcbaGaemyAaKMaemOAaOgabeaakiabg2da9maalaaabaacbiGae8xBa02aaSbaaSqaaiabdMgaPjabdQgaQbqabaGccqGHsislcuWFTbqBgaqbamaaBaaaleaacqWGPbqAcqWGQbGAaeqaaaGcbaGae83Cam3aaSbaaSqaaiabdMgaPjabdQgaQbqabaGccqGHRaWkcuWFZbWCgaqbamaaBaaaleaacqWGPbqAcqWGQbGAaeqaaaaakiaaxMaacaWLjaWaaeWaaeaacqaI1aqnaiaawIcacaGLPaaaaaa@492A@

where *m*_*ij *_is the mean value of the *i*^th ^gene in the *j*^th ^class; *m'*_*ij *_is the mean value of the *i*^th ^gene in all other classes; *s*_*ij *_is the standard deviation of values of the *i*^th ^gene in the *j*^th ^class; *s'*_*ik *_is the standard deviation of values of the *i*^th ^gene in all other classes. (Note the similarity of this metric with the standard two-sample *t*-statistic, T=(m1−m2)/s12/n1+s22/n2
 MathType@MTEF@5@5@+=feaafiart1ev1aaatCvAUfKttLearuWrP9MDH5MBPbIqV92AaeXatLxBI9gBaebbnrfifHhDYfgasaacH8akY=wiFfYdH8Gipec8Eeeu0xXdbba9frFj0=OqFfea0dXdd9vqai=hGuQ8kuc9pgc9s8qqaq=dirpe0xb9q8qiLsFr0=vr0=vr0dc8meaabaqaciaacaGaaeqabaqabeGadaaakeaacqWGubavcqGH9aqpcqGGOaakcqWGTbqBdaWgaaWcbaGaeGymaedabeaakiabgkHiTiabd2gaTnaaBaaaleaacqaIYaGmaeqaaOGaeiykaKIaei4la8YaaOaaaeaacqWGZbWCdaqhaaWcbaGaeGymaedabaGaeGOmaidaaOGaei4la8IaemOBa42aaSbaaSqaaiabigdaXaqabaGccqGHRaWkcqWGZbWCdaqhaaWcbaGaeGOmaidabaGaeGOmaidaaOGaei4la8IaemOBa42aaSbaaSqaaiabikdaYaqabaaabeaaaaa@465A@, where *n *represents the number of cases in a class, *m *is the mean and *s*^2 ^is the variance.)

#### Data sampling strategies

The NCI60 data [1] set is pre-processed using PCA, and the 23 first 'eigengenes' (explaining > 75% of the total variance), are selected. The dimensions of the data set are thus *n *= 60 cases, *p *= 23 features. The data set comprises nine classes. The data set is analyzed in ten-fold repeated random subsampling (a.k.a. *repeated hold-out method*). The ten data set pairs (*L*_*i*_, *T*_*i*_), *i *= 1..10, are generated by randomly sampling 45 (75%) cases for *L*_*i *_and 15 (25%) cases for *T*_*i*_.

For both the acute lymphoblastic leukemia (ALL) data set [3] (*n *= 327 cases, *p *= 12,600 genes, ten classes) and the Global Cancer Map (GCM) data set [2] (*n *= 198 cases, *p *= 16,063 genes, 14 classes), we apply the S2N metric for feature selection. For the weight of each gene, a *p*-value is derived, corresponding to the probability that this weight is obtained by chance alone. The Monte Carlo method to compute this *p*-value involves 1,000 random permutations of the class labels and a recomputation of the weight for each gene [29]. Feature weighting is performed only on the learning set and *not *on the test set.

In contrast to the original study by Yeoh *et al*. [3], the present study investigates whether the less distinct classes (*Hyperdiploid*, *Hypodiploid*, *Pseudodiploid*, and *Normaldiploid*) in the group *Others *show an expression signature that could be used for classification. This implies that instead of merging these subgroups into one single group, these four subgroups are treated as distinct groups. From the pre-processed, normalized data set, we randomly select 215 cases (65.75%) for the learning and 112 cases (34.25%) for the test set. Then, based on the learning set only, we determine the signal-to-noise weight for each gene with respect to each class. We randomly permute the class labels and perform a random permutation test to assess the importance of the signal-to-noise weights [29]. We rank the genes according to their weight and the associated *p*-value; the smaller the *p*-value and the larger the weight, the more important is the gene. We repeat this procedure ten times to generate ten pairs, each consisting of a learning set *L*_*i *_and a test set *T*_*i*_. The models are then built on the learning set *L*_*i *_and tested on the corresponding test set, *T*_*i*_.

**Figure 4:**
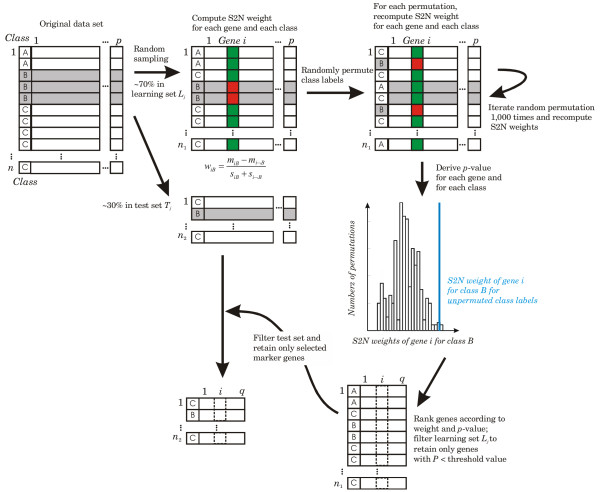
**Sampling of learning and test set and selection of marker genes**. Depicted is one fold in the ten-fold resampling procedure. From the original data set comprising *n *cases and *p *genes, ~70% of the cases are randomly selected for the learning set *L*_*i *_and ~30% cases for the test set *T*_*i*_. On the learning set *L*_*i *_with unpermuted class labels, the signal-to-noise weight for each gene and each class is computed as illustrated for class *B*. The class labels are then randomly permuted 1,000 times and the signal-to-noise weights (for each gene and each class) are recomputed for each permutation to assess the significance of the weights for the unpermuted learning set. Both the learning and the test set are filtered to contain only those genes that are significantly differently expressed in the learning set.

The sampled learning and test sets from the GCM data set are generated as described for the ALL data set. The GCM learning sets include 150 (75.8%) randomly selected cases and the test sets include 48 (24.2%) cases. For each learning set, potential marker genes are identified using signal-to-noise metric in combination with a random permutation test. Figure 4 illustrates the feature selection process that applies to both the ALL and the GCM data set; depicted is only one fold in the tenfold sampling procedure.

In addition to the statistical evaluation, we carried out an epistemological validation to verify whether the identified marker genes are known or hypothesized to be associated with the phenotype under investigation. For example, the majority of the top-ranking genes in the GCM data set could be confirmed to be either known or hypothesized marker genes. In *L*_1_, for instance, the top gene (S2N of 2.84, *P *< 0.01) for the class *colon cancer *is Galectin-4, which is known to be involved in colorectal carcinogenesis [30].

In contrast, the biological interpretation of the 'eigengenes' resulting from PCA is not trivial. We decided not to apply S2N to the NCI60 data set due to the small number of cases (60) and the relatively large number of classes (9). Since feature selection must be performed in each cross-validation fold, it would be necessary to compute the S2N weight for each gene and each class based on each *L*_*i *_comprising only 45 cases, and the computed values for the mean and standard deviation can be highly affected by those cases that are left out for the test set.

All models are trained in leave-one-out cross-validatio*n *(LOOCV) on the learning set *L*_*i *_to determine those parameters that lead to the smallest cumulative error. The models then use these parameters to classify the test cases in *T*_*i*_. Each learning phase encompasses a complete re-calibration of the models' parameters.

### Classifiers

#### Distance-weighted *k*-nearest neighbor classifier

The similarity between two cases, **x**_*i *_and **x**_*j*_, is commonly defined as

*similarity*(**x**_*i*_, **x**_*j*_) = 1 - *distance*(**x**_*i*_, **x**_*j*_)     (6)

A *k*-NN classifier can be based on simple majority voting that takes into account only the classes and their frequencies in the set of *k*_*opt *_nearest neighbors (ignoring their similarity with the test case). A yet more sophisticated incarnation of the *k*-NN classifier takes into account how similar the respective nearest neighbors are to the test case. These similarity scores are then used to calculate a confidence value in a weighted voting scheme.

The *k*-NN in this study operates as follows. Let **n**_*k *_denote the *k*^th ^nearest neighbor of a test case **x**_*j *_and the optimal number of nearest neighbors be *k*_*opt*_. Further, let the similarity, *sim*, between cases **x**_*i *_and **x**_*j *_be given by 1 - *d*(**x**_*i*_, **x**_*j*_), where *d *represents a distance. In the present study, we investigate various distance metrics, including Euclidean, Canberra, Manhattan, and the fractional distance [31]. The *normed similarity *between **x**_*j *_and its nearest neighbor **n**_*k*_, *sim*_*normed*_(**x**_*j*_, **n**_*k*_), is then defined as

simnormed(xj,nk)=sim(xj,nk)∑k=1koptsim(xj,nk)     (7)
 MathType@MTEF@5@5@+=feaafiart1ev1aaatCvAUfKttLearuWrP9MDH5MBPbIqV92AaeXatLxBI9gBaebbnrfifHhDYfgasaacH8akY=wiFfYdH8Gipec8Eeeu0xXdbba9frFj0=OqFfea0dXdd9vqai=hGuQ8kuc9pgc9s8qqaq=dirpe0xb9q8qiLsFr0=vr0=vr0dc8meaabaqaciaacaGaaeqabaqabeGadaaakeaacqWGZbWCcqWGPbqAcqWGTbqBdaWgaaWcbaGaemOBa4Maem4Ba8MaemOCaiNaemyBa0MaemyzauMaemizaqgabeaakiabcIcaOiabhIha4naaBaaaleaacqWGQbGAaeqaaOGaeiilaWIaeCOBa42aaSbaaSqaaiabdUgaRbqabaGccqGGPaqkcqGH9aqpdaWcaaqaaiabdohaZjabdMgaPjabd2gaTjabcIcaOiabhIha4naaBaaaleaacqWGQbGAaeqaaOGaeiilaWIaeCOBa42aaSbaaSqaaiabdUgaRbqabaGccqGGPaqkaeaadaaeWaqaaiabdohaZjabdMgaPjabd2gaTbWcbaGaem4AaSMaeyypa0JaeGymaedabaGaem4AaS2aaSbaaWqaaiabd+gaVjabdchaWjabdsha0bqabaaaniabggHiLdGccqGGOaakcqWH4baEdaWgaaWcbaGaemOAaOgabeaakiabcYcaSiabh6gaUnaaBaaaleaacqWGRbWAaeqaaOGaeiykaKcaaiaaxMaacaWLjaWaaeWaaeaacqaI3aWnaiaawIcacaGLPaaaaaa@6B96@

The degree of class membership is then defined as follows:

p^(C|xj)=∑k=1koptδksimnormed(xj,nk)     (8)
 MathType@MTEF@5@5@+=feaafiart1ev1aaatCvAUfKttLearuWrP9MDH5MBPbIqV92AaeXatLxBI9gBaebbnrfifHhDYfgasaacH8akY=wiFfYdH8Gipec8Eeeu0xXdbba9frFj0=OqFfea0dXdd9vqai=hGuQ8kuc9pgc9s8qqaq=dirpe0xb9q8qiLsFr0=vr0=vr0dc8meaabaqaciaacaGaaeqabaqabeGadaaakeaacuWGWbaCgaqcaiabcIcaOiabdoeadjabcYha8jabhIha4naaBaaaleaacqWGQbGAaeqaaOGaeiykaKIaeyypa0ZaaabCaeaaiiGacqWF0oazdaWgaaWcbaGaem4AaSgabeaakiabdohaZjabdMgaPjabd2gaTnaaBaaaleaacqWGUbGBcqWGVbWBcqWGYbGCcqWGTbqBcqWGLbqzcqWGKbazaeqaaaqaaiabdUgaRjabg2da9iabigdaXaqaaiabdUgaRnaaBaaameaacqWGVbWBcqWGWbaCcqWG0baDaeqaaaqdcqGHris5aOGaeiikaGIaeCiEaG3aaSbaaSqaaiabdQgaQbqabaGccqGGSaalcqWHUbGBdaWgaaWcbaGaem4AaSgabeaakiabcMcaPiaaxMaacaWLjaWaaeWaaeaacqaI4aaoaiaawIcacaGLPaaaaaa@5E24@

where the Kronecker symbol *δ*_*k *_= 1 if **n**_*k *_∈ *C *and *δ*_*k *_= 0 otherwise. If a crisp classification is required, then a case **x**_*j *_may be classified as member of class *C *for which p^
 MathType@MTEF@5@5@+=feaafiart1ev1aaatCvAUfKttLearuWrP9MDH5MBPbIqV92AaeXatLxBI9gBaebbnrfifHhDYfgasaacH8akY=wiFfYdH8Gipec8Eeeu0xXdbba9frFj0=OqFfea0dXdd9vqai=hGuQ8kuc9pgc9s8qqaq=dirpe0xb9q8qiLsFr0=vr0=vr0dc8meaabaqaciaacaGaaeqabaqabeGadaaakeaacuWGWbaCgaqcaaaa@2E25@ (*C *| **x**_*j*_) is maximal.

**Figure 5:**
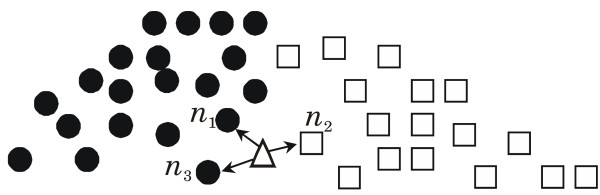
**The distance-weighted *k*-NN classifier for a binary classification task**. The arrows indicate the three nearest neighbors of the test case. Here it is assumed that *k*_*opt *_= 3.

Figure 5 illustrates the *k*-NN on a simplified example involving only two classes. In this example, the triangle marks the test case.

Table 3 shows the derived scores of class membership and the classification result.

**Table 3:** The distance-weighted *k*-NN for the example data shown in Figure 5.

*Nearest neighbor*	*Similarity*	*sim*_*normed*_	*Predicted class*
**n**_1_	0.09	0.36	●
**n**_2_	0.08	0.32	□
**n**_3_	0.08	0.32	●

The degree of class membership for class • is then 0.36 + 0.32 = 0.68, and the degree for class □ is 0.32. Note that in contrast to 'classic' *k*-NN models, the proposed model allows for the rejection of low-confidence classifications. The classification implies that the example test case is a member of class • with a degree of 0.68 and a member of class □ with a degree of 0.32.

#### 'Classic' nearest neighbor classifiers: 1-NN, 3-NN, and 5-NN

The 1-NN is the simplest implementation of an instance-based learner, which assigns to a test case the same class as the most similar case in the learning set. The 3-NN and 5-NN classifiers retrieve three (five) nearest neighbors and assign the majority class to the test case. If no majority class exists (for example, if the 3-NN retrieves neighbors of three different classes or if the 5-NN retrieves two cases of class *A*, two cases of class *B *and one case of class *C*), then the classifiers retrieve the next nearest neighbor until the tie is broken.

#### Support vector machines

The support vector machines [32] in the present study implement three different kernel functions: Linear kernel *K*(**x**_*i*_, **x**_*j*_) = (**x**_*i*_·**x**_*j*_), radial kernel *K*(**x**_*i*_, **x**_*j*_) = exp(-||**x**_*i *_- **x**_*j*_||^2 ^/ 2*σ*^2^), and the polynomial kernel *K*(**x**_*i*_, **x**_*j*_) = (**x**_*i*_·**x**_*j *_+ 1)^*d*^, with *d *= 2 or *d *= 3. For the present study we used the implementation from [33].

SVMs are inherently binary classifiers, and it is not obvious how they can solve problems that comprise more than two classes. There exist two commonly adopted approaches for breaking down multiclass problems into a sequence of binary problems: (*i*) the *one-versus-all *(OVA) approach, and (*ii*) the *all-pairs *(AP) approach. For the present study, we combined the SVMs in the AP approach, which constructs 1\2 *k*(*k *- 1) classifiers, with each classifier trained to discriminate between a class pair *i *and *j*. The outputs of the binary classifiers are then combined in a *decision directed acyclic graph *(DDAG), which is a graph whose edges have an orientation and no cycles [34]. Mukherjee pointed out that the decision boundaries resulting from the all-pairs approach are, in general, more natural and intuitive, and should be more accurate in theory [35]. For the present study, we combined the SVMs in the AP approach. The SVMs are trained in LOOCV on the learning set to determine the optimal parameters, i.e., the optimal kernel function, the optimal kernel parameters (bandwidth for the Gaussian kernel and the degree of the polynomial kernel), and the optimal error penalty.

#### Decision tree C5.0

The term 'decision tree' is derived from the presentation of the resulting model as a tree-like structure. Decision tree learning follows a top-down, divide-and-conquer strategy. The basic algorithm for 'decision tree learning' can be described as follows [36]:

(1) Select (based on some measure of 'purity' or 'order' such as *entropy*, *information gain*, or *diversity*) an attribute to place at the root of the tree and branch for each possible value of the tree. This splits up the underlying case set into subsets, one for every value of the considered attribute.

(2) *Tree growing*: Recursively repeat this process for each branch, using only those cases that actually reach that branch. If at any time most instances at a node have the same classification or if a further splitting does not lead to a significant improvement, then stop developing that part of the tree.

(3) *Tree pruning*: Merge some nodes to improve the model's performance, i.e., balance the bias and variance of the tree based on statistical measures regarding the node purity or based on performance assessment (e.g., cross-validation performance).

Following the top-down and divide-and-conquer strategy, learning in C5.0 involves a tree growing phase and a tree pruning phase. In the pruning phase some nodes are merged to improve the generalization ability of the overall model. C5.0 builds a multi-leaf classification tree based on information gain ranking of the attributes.

The initial pruning severity of the decision tree is 90%. Then, in 10-fold cross-validation on the learning set, the average correct classification rate is determined. The pruning severity is iteratively reduced in steps of 10% (i.e., 90%, 80%, 70% etc.), and the tree is rebuilt in 10-fold cross-validation. Using this strategy, the optimal pruning severity is determined for the learning set. The DT is then built on the entire learning set *L*_*i *_and pruned with the optimal pruning severity. The resulting model is used to classify the corresponding test cases in *T*_*i*_.

#### Multilayer perceptrons

For both the decision tree and the multilayer perceptrons, SPSS Clementine's^® ^implementation is used. Various network topologies are investigated in the present study; the optimal architecture (number of layers and hidden neurons) is determined in the learning phase. The training algorithm for the multilayer perceptrons is backpropagation with momentum *α *= 0.9 and adaptive learning rate of initial λ = 0.3. The network is initialized with one hidden layer comprising five neurons. The number of hidden neurons is empirically adapted on the learning set *L*_*i*_, i.e., the network topology is chosen to provide for the lowest cross-validated error rate on the learning set *L*_*i*_. The resulting optimal network architecture is chosen for predicting the test cases in *T*_*i*_.

## Authors' contributions

DB implemented the NN models, selected and pre-processed the data sets, and carried out the comparative study. IB helped in the statistical design and interpretation. WD interpreted the results and helped in the preparation of the manuscript.
